# Evaluation of CD46 re-targeted adenoviral vectors for clinical ovarian cancer intraperitoneal therapy

**DOI:** 10.1038/cgt.2016.22

**Published:** 2016-05-27

**Authors:** S L Hulin-Curtis, H Uusi-Kerttula, R Jones, L Hanna, J D Chester, A L Parker

**Affiliations:** 1 Division of Cancer and Genetics, Cardiff University School of Medicine, Cardiff, UK; 2 Velindre Cancer Centre, Cardiff, UK

## Abstract

Ovarian cancer accounts for >140 000 deaths globally each year. Typically, disease is asymptomatic until an advanced, incurable stage. Although response to cytotoxic chemotherapy is frequently observed, resistance to conventional platinum-based therapies develop rapidly. Improved treatments are therefore urgently required. Virotherapy offers great potential for ovarian cancer, where the application of local, intraperitoneal delivery circumvents some of the limitations of intravenous strategies. To develop effective, adenovirus (Ad)-based platforms for ovarian cancer, we profiled the fluid and cellular components of patient ascites for factors known to influence adenoviral transduction. Levels of factor X (FX) and neutralizing antibodies (nAbs) in ascitic fluid were quantified and tumor cells were assessed for the expression of coxsackie virus and adenovirus receptor (CAR) and CD46. We show that clinical ascites contains significant levels of FX but consistently high CD46 expression. We therefore evaluated *in vitro* the relative transduction of epithelial ovarian cancers (EOCs) by Ad5 (via CAR) and Ad5 pseudotyped with the fiber of Ad35 (Ad5T*F35++) via CD46. Ad5T*F35++ achieved significantly increased transduction in comparison to Ad5 (*P*<0.001), independent of FX and nAb levels. We therefore propose selective transduction of CD46 over-expressing EOCs using re-targeted, Ad35-pseudotyped Ad vectors may represent a promising virotherapy for ovarian cancer.

## Introduction

Ovarian cancer is the seventh most common cancer in women worldwide, with nearly 239 000 new cases diagnosed in 2012 (http://www.cancerresearchuk.org/health-professional/ovarian-cancer-statistics). The 5-year survival rates for ovarian cancer are poor and have remained largely unchanged over the past 25 years. This is due in part to the anatomical location of the ovaries, deep within the pelvis contributing to an asymptomatic disease and consequent late diagnosis.^1^ Although response to cytotoxic chemotherapy is frequently observed, resistance to conventional platinum-based therapies develop rapidly. New therapies for relapsed, metastasized ovarian cancer are therefore urgently required.

Ovarian cancer represents a potential candidate for virotherapy, as local delivery to tumor metastases via the intraperitoneal route is feasible, bypassing many of the requirements associated with delivery via the bloodstream. Adenoviruses (Ads) have been widely studied as promising new therapeutic agents for the treatment of a variety of cancers.^2^ Of the 57 naturally occuring different serotypes of human Ads, only those based on the species C Ad5 have been extensively studied for virotherapeutic clinical applications. Ad5 is clinically and experimentally well characterized, readily manipulated by genetic and chemical modification and is easy to amplify to high titers of clinical-grade purity. To date, however, even as adjunctive therapies the efficacy of Ad5 virotherapies have been modest and has been hampered by several limitations that ultimately limit Ad efficacy.^3, 4^ These include a lack of selectivity to infect disease target cell types,^5^ neutralization by preexisting host-neutralizing antibodies (nAbs) of the humoral immune system and sequestration within non-target tissues. This occurs via interactions with host proteins involved in blood clotting, (especially the liver, where Ad is cleared).^6, 7^

Ad5 efficacy is partly dependent on target cell entry via the native primary receptor coxsackie virus and adenovirus receptor (CAR).^8^ However, CAR expression is commonly downregulated in many advanced cancers, including ovarian cancer,^9, 10, 11^ limiting the clinical utility of Ad5-based vectors. Genetic and chemical modification of Ad5, which has been de-targeted from the CAR receptor but with re-targeted tropism toward alternative, differentially expressed receptors, has been well studied^12, 13^ and represents a strategy for effective, targeted virotherapies.

Ad5 commonly causes upper respiratory and gastrointestinal infections and thus preexisting nAbs are widespread in the population.^14^ This results in rapid, efficient sequestration of systemically delivered Ad5 vectors *in vivo*.^3, 4^ Ascites, an accumulation of fluid within the patient's abdomen, is a common clinical feature of ovarian cancer and a reservoir for nAbs. The presence of nAbs represents a barrier to the efficacy of unmodified Ad5-based virotherapies for peritoneal tumor deposits when delivered via intraperitoneal installation.^15^ Conversely, nAbs to the rarer, species B serotype Ad35 are seen in typically <10% of the population.^14, 16, 17^ In case of kidney and urinary tract infections and conjunctivitis,^18^ cell transduction by Ad35 is via the CD46 receptor,^19^ which is expressed on almost all nucleated cells and commonly upregulated in cancer.^20^ Previous studies have explored the possibility of pseudotyping the Ad5 vector with fibers from group B Ads for improved gene delivery via the CD46 pathway^21^ and Ad35-based vectors^22^ have shown great promise for gene transfer to a variety of cancer cells.^23, 24^

The presence of blood coagulation factor X (FX) is a significant obstacle to the efficacy of Ad-mediated virotherapy.^6, 7^ Following intravascular administration, the Ad5 hexon hypervariable regions bind FX with very high affinity, forming a complex of virus and clotting factor with heparan sulfate proteoglycan (HSPGs), which is found on many cell types but in high abundance on hepatocytes. The presence of FX in ascites could limit the transduction of epithelial ovarian cancer (EOC) cells, either via facilitating entry into non-target cells expressing HSPGs or, where no cells expressing HSPGs are present, via steric hindrance of CAR-mediated cell entry into tumor cells.

We hypothesized that the use of an Ad5-based vector with ablated FX binding and pseudotyped with the Ad35 fiber (Ad5T*F35++) with a 60-fold increased affinity for CD46^25^ will reduce interactions with off-target cells and enhance EOC transduction.

## Materials and methods

### Ethics approval

Ethics permission for the collection and cultivation of primary EOC cells from ascites was granted through a Wales Cancer Bank application for biomaterials, reference WCB 14/004. All patients gave written informed consent for the use of their samples, prior to collection.

### Generation of Ad vectors

Ad5.Luc vector was generated by AdZ homologous recombineering as previously described.^26^ The Ad5 LacZ vector incorporating a mutation in the hexon variable region (HVR7) and pseudotyped with the Ad35 fiber Ad5CMV-HCR5*7*E451Q/F35++ (herein referred to as Ad5T*F35++) was generated previously^27^ and was a kind gift from Professor Andrew Baker (BHF Glasgow Cardiovascular Research Centre, Glasgow University, Glasgow, UK).

### Primary EOC cells

Ascites samples were collected from a total of 11 patients with varying clinical stages of ovarian cancer (stage diagnosed at sample collection) at the Velindre Cancer Centre, Cardiff, UK and anonymously coded. Ascites was stored at 4 °C immediately after collection and processed within 24 h. Approximately 400 ml of ascites was centrifuged at 1000 r.p.m. for 5 min to separate primary EOC cells from the fluid. The supernatant was stored at −70 °C for subsequent use with autologous tumor cells. Red blood cell lysis buffer (Sigma Aldrich, Gillingham, UK) was added to the pellet according to the manufacturer's instructions, where appropriate. Tumor cell pellets were frozen in 10% dimethyl sulfoxide and 90% autologous supernatant (passage 0). A further 100 ml of ascites was used to generate primary EOC cultures, by separating into 20-ml aliquots and adding to 20 ml of complete (RPMI 1640) medium, supplemented with 10% (v/v) fetal calf serum, 200 μm glutamine, 100 U ml^−1^ penicillin, 100 μg ml^−1^ streptomycin and 10% (v/v) autologous ascitic fluid supernatant. Cells were maintained at 37 °C and 5% CO_2_. The resulting primary cultures were passaged when cells had reached confluence.

### Cell lines

A549 (epithelial lung carcinoma cells) in RPMI 1640 medium, both supplemented with 10% fetal calf serum, 2 mm L-glutamine, 100 U ml^−1^ penicillin and 100 μg ml^−1^ streptomycin. Cells were maintained at 37 °C and 5% CO_2_. All reagents were purchased from Gibco or Thermo Scientific (Paisley, UK).

### Flow cytometric analysis of primary EOC cell receptor expression

Receptor expression was profiled essentially as previously described.^15^ In brief, EOC cells (1.5 × 10^5^ cells per well in a 96-well plate) were seeded and washed in 200 μl of wash buffer (phosphate-buffered saline/1% bovine serum albumin) and incubated with 100 μl of wash buffer containing 1:500 of mouse anti-human monoclonal antibody against CAR (RmcB, Millipore, Watford, UK), 1:500 of mouse anti-human CD46 (MEM-258, Abcam, Cambridge, UK) or mouse immunoglobulin G control antibody (Santa Cruz Biotechnology, Heidelberg, Germany) for 1 h on ice. Cells were washed three times and incubated with a 1:500 dilution of goat anti-mouse Alexa Fluor 647 antibody (Invitrogen, Paisley, UK) for 1 h on ice. Cells were fixed in 4% paraformaldehyde for a minimum of 10 min at 4 °C for flow cytometry. In all, 2 × 10^4^ gated events were acquired in channel FL-4 on a BD Accuri C6 (BD Biosciences, San Jose, CA, USA) flow cytometer and data were analyzed in the BD Accuri C6 software version 1.0.264.21 (Becton Dickinson, Franklin Lakes, NJ, USA).

### FX enzyme-linked immunosorbent assay

The quantity of FX in cell-free ascites from each of 11 ovarian cancer patients was determined using the Factor X Human ELISA Kit (Abcam) according to the manufacturer's instructions with a Bio-Rad iMark microplate reader (Bio-Rad, Hemel Hempstead, UK).

### *In vitro* cell transduction assays

Assays were performed as previously described.^15^ In brief, cells were seeded at a density of 2 × 10^4^ cells per well in a 96-well plate. After 24 h, cells were infected with virus at doses of 5000 and 10 000 virus particles (vp) per cell in a total volume of 100 μl of serum-free medium and incubated as above for 3 h. The medium was removed and replaced with 200 μl of complete medium (RPMI 1640 medium supplemented with 200 μm Glutamax, 10% (v/v) fetal calf serum, 100 U ml^−1^ penicillin, 100 μg ml^−1^ streptomycin and 10% (v/v) autologous supernatant) and cultured for an additional 45 h. For luciferase assays, cells treated with Ad5.Luc were lysed in 1 × Cell Culture Lysis Buffer (Promega, Southampton, UK) and frozen at −70 °C. The cells were thawed and 20 μl of cells was mixed with 100 μl of luciferase assay reagent in a white 96-well plate. Luciferase activity in relative light units (RLU) was measured immediately using a multimode plate reader (FLUOstar Omega, BMG Labtech, Aylesbury, UK). Samples were normalized for total protein content, as measured by bicinchoninic acid assay in RLU per mg protein. For cells transduced with the LacZ-containing Ad5T*F35++ vector, cells were lysed in β-Galactosidase lysis buffer (Galacto-Light Plus Systems Chemiluminescent Reporter Gene Assay System for the Detection of β-Galactosidase, Applied Biosystems, Waltham, MA, USA) and frozen at −70 °C. Cells were thawed and 10 μl of cells mixed with 70 μl of β-Galactosidase reactant (1:100 Galacton-Plus and β-Galactosidase diluent, Applied Biosystems). Cells were incubated at room temperature for 1 h and 100 μl of Tropix Accelerator II (Applied Biosystems) was added to cells immediately prior to measurement of β-Galactosidase activity. β-Galactosidase activity was measured in RLU using the plate reader as described above. Samples were normalized for total protein content as measured by bicinchoninic acid assay in RLU per mg protein, using the plate reader as described above.

### Cell transduction in the presence of FX and CD46 function blocking

Cells were transduced as described above in either the presence or absence of 10 μg ml^−1^ of human FX (Haematologic Technologies, Cambridge Bioscience, Cambridge, UK) or mouse anti-human anti-CD46 (MEM-258) antibody (Abcam), respectively. Mouse immunoglobulin G antibody (Santa Cruz Biotechnology, Heidelberg, Germany) was used as a control.

### Ascitic fluid neutralization assay

A549 lung carcinoma cells were seeded at 2 × 10^4^ cells per well in a 96-well plate. Cells were infected with virus at a dose of 5000 vp per cell in serum-free media together with a 1:40 dilution (2.5%) of supernatant derived from ascites that contains nAbs. Cells were transduced as described above.

### Statistical analyses

Results represent data expressed as the mean±s.e.m. from experiments performed in triplicate. Differences in the number of ascites samples used for experiments was due to limited availability of samples. Statistical significance was calculated using two-sample, two-tailed *t*-tests (Excel software, Microsoft Ltd, Reading, UK). *P*<0.05 was considered statistically significant.

## Results

### Expression of Ad receptors on EOC cells

**Figure 1:**
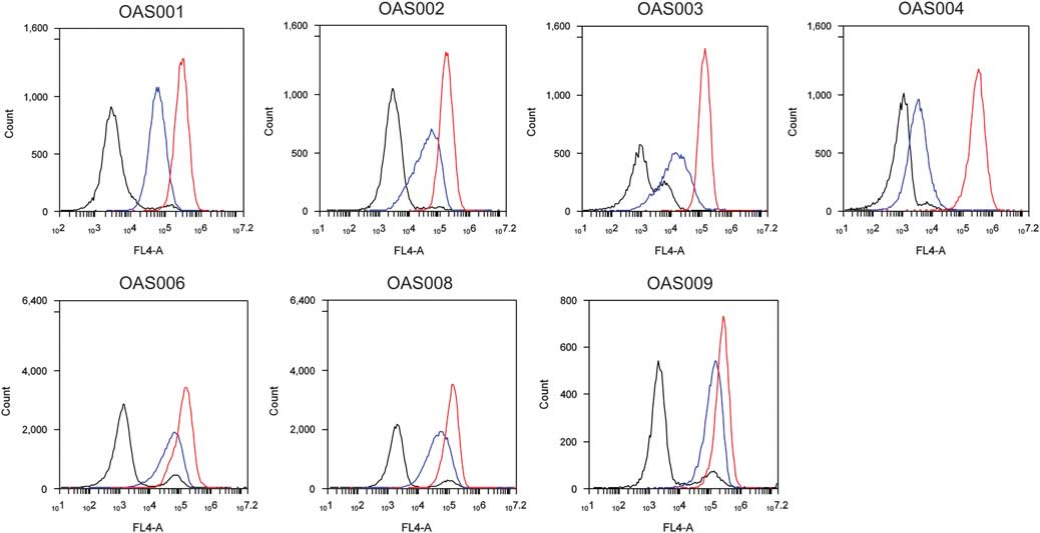
Flow cytometric plots showing the expression profiles of coxsackie and adenovirus receptor (CAR) and CD46 expression on primary epithelial ovarian cancer (EOC) cells. Primary EOC cells were cultured *ex vivo* from seven ovarian cancer patients and stained for the expression of CAR (red) and CD46 (blue) using mouse anti-human monoclonal antibody against CAR or CD46 (anti-CD46 antibody, MEM-258) as determined by flow cytometry. Immunoglobulin G controls are shown in black.

Expression profiles of the native Ad5 receptor CAR and the Ad35 receptor CD46 were characterized on primary EOC cells cultured from the ascites of seven ovarian cancer patients (Figure 1). Primary EOC cells demonstrated variable CAR expression, ranging from low (30%) to high (99%) expression, while CD46 was expressed at high levels in all samples (Table 1).

### Analysis of levels of FX in clinical ascites samples

**Figure 2:**
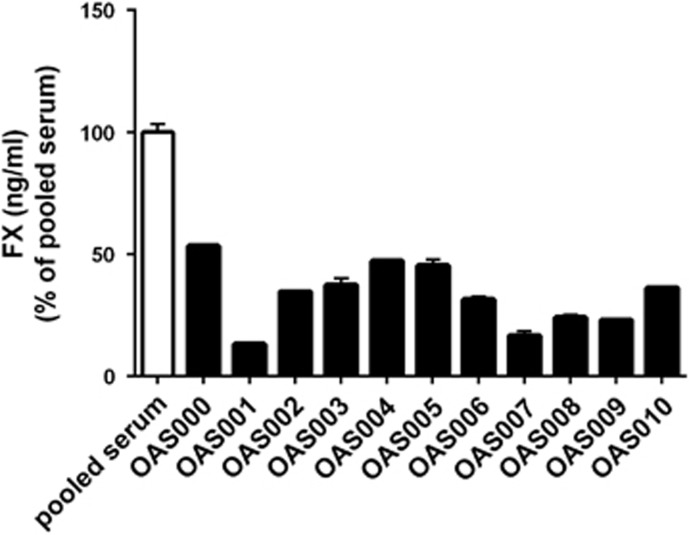
Factor X (FX) is present in significant levels in the ascites fluid derived from ovarian cancer patient clinical isolates. The ascites fluid from six ovarian cancer patients was assayed for coagulation FX by enzyme-linked immunosorbent assay.

FX has previously been shown to limit the bioavailability of Ad5 in the bloodstream and redirect viral tropism via HSPGs. We sought to establish whether FX could be detected in the ascites from patients with ovarian cancer by enzyme-linked immunosorbent assay, to establish whether FX might be a potential barrier to Ad transduction of tumor cells when delivered intraperitoneally. We were able to detect significant levels of FX present in all the samples tested, with concentrations varying from 13% to 53% of levels seen in normal (pooled) serum (*n*=5) (Figure 2).

### EOC cell transduction by pseudotyped Ad5T*F35++ vector

**Figure 3:**
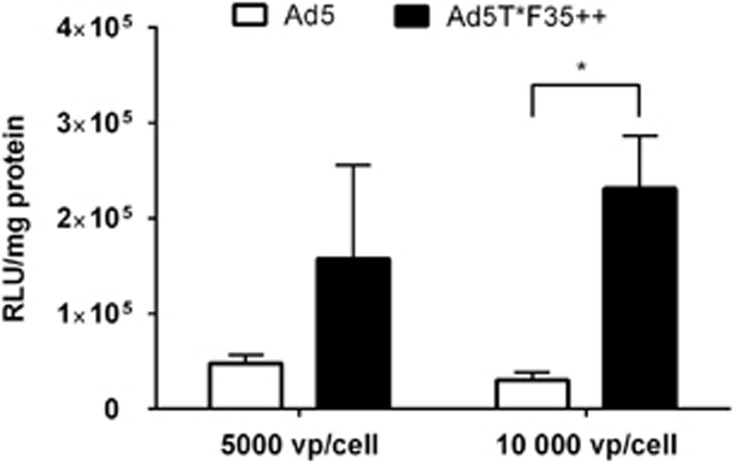
*In vitro* epithelial ovarian cancer (EOC) cell transduction by Ad5T*F35++. Primary EOC cells were infected with Ad5 (luciferase expressing) or Ad5T*F35++ (β-Galactosidase expressing) at 5000 and 10 000 virus particles (vp) per cell. Cell transduction was measured by luciferase and β-Galactosidase activity 48 h postinfection and normalized for protein content by bicinchoninic acid assay. RLU, relative light units. *P<0.05.

The capacity of the parental Ad5 (control) vector and the hexon-mutated, pseudotyped vector Ad5T*F35++ to transduce EOC cells was investigated *in vitro* (Figure 3). We observed an increase in transduction of EOCs by Ad5T*F35++ vector (5000 vp per cell) in comparison to the parental Ad5 vector, although this did not reach statistical significance (*P*=0.06). At 10 000 vp per cell, Ad5T*F35++ transduction was significantly increased by 7.6-fold (*P*<0.001) in comparison to the Ad5 vector.

### CD46 receptor usage by Ad5T*F35++ vector in transducing EOC cells

**Figure 4:**
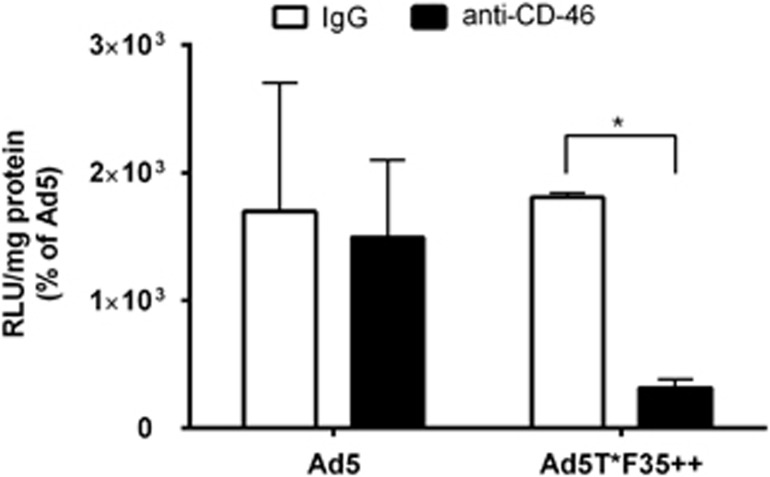
Ad5T*F35++ vector uptake is blocked by anti-CD46 function antibody MEM-258. Primary epithelial ovarian cancer (EOC) cells were infected with Ad5 (luciferase expressing) or Ad5T*35++ (β-galactosidase expressing) at 5000 virus particles per cell in the presence or absence of anti-CD46 (MEM-258) antibody (10 μg ml^−1^). Cell transduction was measured by luciferase and β-Galactosidase activity 48 h postinfection and normalized for protein content by bicinchoninic acid assay. RLU, relative light units. *P<0.05.

To confirm that the Ad5T*F35++ vector transduces EOC cells via the CD46 receptor, we blocked CD46 receptors by preincubating EOC cells with anti-CD46 antibody MEM-258 for 1 h, prior to the addition of the Ad5T*F35++ vector (Figure 4). Our results confirm that cellular transduction of Ad5T*F35++ in EOC cells was significantly reduced (*P*<0.001) in the presence of anti-CD46-blocking antibody.

### Evaluation of Ad vector neutralization in the presence of ascitic fluid

**Figure 5:**
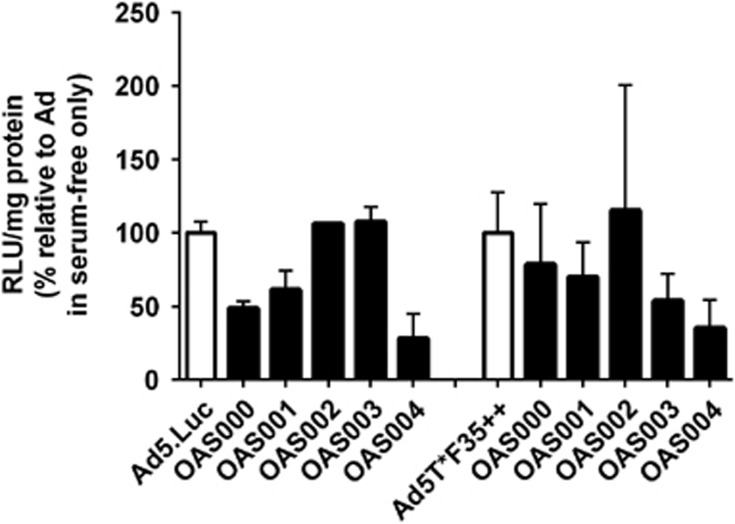
Transduction of the Ad5T*F35++ vector is not significantly neutralized by ascitic fluid. A549 cells were transduced with Ad5 and Ad5T*F35++ vectors (5000 viral particles per cell) in the presence of serum-free media or 2.5% ascitic fluid supernatant from ovarian cancer patients (OAS000–OAS004). Luciferase (Ad5.luc) and β-Galactosidase (Ad5T*F35++) activity was measured 48 h postinfection and normalized for protein content by bicinchoninic acid assay. Transduction (%) is presented normalized to Ad5 and Ad5T*F35++ transduction in serum-free media. RLU, relative light units.

To investigate whether pseudotyping Ad5 with the Ad35 fiber alters the potential for neutralization by nAbs in ascites, Ad5T*F35++ transduction experiments were performed in the presence of 2.5% ascitic fluid (Figure 5). A549 cells were used (CAR^high^/CD46^high^) consistent with previously published protocols. Cell transduction of the Ad5T*F35++ vector in the presence of ascites was not significantly affected implying that FX in the ascitic fluid does not hinder cellular entry of the modified virus.

## Discussion

Ad5 is the most commonly used Ad vector for virotherapy applications. However, downregulated expression of the native Ad5 cell entry receptor CAR in many tumor cells, coupled with the high affinity binding of Ad5 to FX mediates off-target effects that may result in dose-limiting toxicities. Ad vector neutralization by preexisting ascites-resident nAbs^4, 15, 18, 28^ greatly limits the efficacy of Ad5-based vectors for ovarian cancer treatment by intraperitoneal delivery.

The aim of this study was to characterize ascites samples from ovarian cancer patients and the EOC cells derived from these samples in order to develop a rational approach for intraperitoneally delivered virotherapy. To the best of our knowledge, this is the first report of significant levels of FX in the ascites of ovarian cancer patients. This has implications for the clinical potential of intraperitoneal delivery of Ad5 vectors, as Ad5 has the potential for transducing off-target cells owing to its ability to use both CAR and HSPGs (via FX binding) for cell entry and to potentially transduce the liver (should leakage of the vector into the bloodstream occur).

We show that expression of CAR in EOC cells is highly variable, ranging from low (30%) to high (99%) expression, possibly correlating to disease stage, potentially limiting the efficacy of Ad5 vectors for patients whose tumors have low CAR expression. However, CD46 expression was constitutively high on EOC cells derived from all seven ovarian cancer patients in our cohort. To exploit the constitutive high levels of expression of CD46 on EOC cells, we evaluated the targeting potential of the Ad5T*F35++ vector, which is pseudotyped with the Ad35 fiber and therefore potentially able to transduce cells expressing CD46. We demonstrate a significant, CD46-dependent increase in EOC cell transduction with Ad5T*F35++ infection in EOC cells in comparison to the parental Ad5 control vector. This effect is blocked by anti-CD46 antibody but not by ascitic fluid containing FX (and nAbs), confirming that the enhanced transduction achieved by Ad5T*F35++ is achieved via pseudotyping, rather than by ablation of FX binding.

Ad35-based vectors have shown potential for delivery of gene transfer to cancer cells.^23, 24^ Earlier studies report that Ad5-based vectors pseudotyped with the Ad35 fiber show improved infection of colon (HT29) and ovarian (SKOV3) cancer cells^23^ and primary ovarian cancer cells using an oncolytic Ad5/F35 *in vitro*.^29^ Ad35 demonstrated high cytotoxicity in cancer cell lines compared with other group C and B viruses but a lack of oncolytic activity *in vivo*.^30^ This may be context-dependent owing to conditions of the tumor microenvironment and requires further vector optimization. A recent report demonstrated that infections of paclitaxel-resistant ovarian cancer cell models with Ad11 and Ad35 oncolytic Ads were significantly more effective.^31^ Furthermore, pseudotyping with the Ad35 fiber had no effect on *in vitro* transduction in Chinese Hamster Ovary CHO-CD46 cells in the presence of FX.^32^ This is thought to be due to an over-riding effect of high affinity binding of Ad35 with CD46, an effect abrogated in CHO wild-type receptor cells lacking CD46. Others have suggested that Ad5/F35 virus particles accumulate in the late endosome resulting in delayed trafficking to the nucleus (reduced transduction) and exocytosis into the extracellular medium. This suggests that the Ad35 fiber dominates internalization and trafficking, despite hexon and HSPG interactions via FX binding.^33^ Although it is largely accepted that FX:hexon binding dictates liver tropism, it is reported that, upon viral cell entry, this interaction may also activate the host immune system via the Toll-like receptor 4/tumor necrosis factor receptor-associated factor 6/nuclear factor-ĸB pathway.^34^ On the other hand, a report has suggested that ‘coating' of Ad by FX actually shields the Ad from immune-mediated neutralization *in vivo* and therefore represents a protective mechanism for preserving the Ad.^35^ These studies are controversial and may reflect context-dependent tumor microenvironments. However, this highlights the potential clinical utility of Ad35 fiber-pseudotyped vectors.

We previously reported a significant decrease in Ad5 EOC cell transduction in the presence of ascitic fluid owing to preexisting nAbs and show that genetic modification of the Ad5 fiber knob can facilitate evasion of nAbs in some patient samples.^15^ Others have demonstrated that approximately 33% of serum samples from patients undergoing coronary artery bypass graft surgery reduced Ad5 transduction by at least 90%, whereas Ad5T*F35++ transduction was neutralized by only 18% of their serum samples.^36^ In this study, we evaluated the transduction capability of the Ad5T*F35++ vector that does not bind FX, for consideration as a candidate virotherapy for intraperitoneal delivery, circumventing the limitations associated with systemic delivery. We performed cell transduction experiments in A549 cells that express high levels of CAR and CD46.^36^ Collectively, our data show no difference in neutralization of the Ad5T*F35++ vector in the presence of 2.5% ascitic fluid in comparison to the Ad5 parental vector.

In summary, we demonstrate that ascites of ovarian cancer patients at various stages of the disease contains significant levels of FX, precluding the use of the Ad5 vector for intraperitoneal Ad delivery. EOC cells derived from the ascites show variable expression of CAR but ubiquitous expression of the Ad35 receptor CD46, suggesting that pseudotyping with Ad35 fibers may enhance the efficiency of viral transduction. The FX binding-ablated, re-targeted Ad5T*F35++ vector presumed to use the CD46 receptor for cell entry instead of CAR demonstrated significantly increased EOC cell transduction in comparison to the parental Ad5 vector. There was blocking of this effect by anti-CD46 antibody but no significant neutralization of the Ad5T*F35++ vector incubated in the presence of ascitic fluid. This study demonstrates the Ad5T*F35++ vector as a potential virotherapy for ovarian cancer and warrants further investigation.

## Figures and Tables

**Table 1:** Characterization of *ex vivo* primary epithelial ovarian cancer cell receptor expression (%)

*Patient ID*	*Disease stage*	*CAR*	*CD46*
OAS001	2	95	100
OAS002	3	67	100
OAS003	1	40	100
OAS004	4	30	100
OAS006		98	100
OAS008	3	92	100
OAS009	1	99	99

Abbreviation: CAR, coxsackie virus and adenovirus receptor.
